# Analysis of related factors of scheduled ICU before primary hip arthroplasty

**DOI:** 10.1186/s12871-022-01737-y

**Published:** 2022-07-13

**Authors:** Jianguang Sun, Yali Yang, Guilan Feng, Chuanxing Liang, Weiming Ling, Hongxin Liao

**Affiliations:** Joint Surgery of Meizhou People’ S Hospital, Meizhou, 514000 Guangdong Province China

**Keywords:** ICU reservation, Primary hip arthroplasty, Relative factors analysis

## Abstract

**Background:**

Methods for improving the safety of medical treatments for patients, reducing the occurrence of postoperative complications and optimizing medical resources for primary hip replacement are needed. Previous literature has mostly analysed the risk factors and constructed and models to predict a transfer to the ICU after surgery, and no reports on preoperative ICU reservations have been found. This study evaluated the risk factors for preoperative ICU reservation and considered the necessity of preoperative ICU reservations to optimize preoperative communication, enable a seamless transfer between the operating room and ICU, reduce postoperative complications and shorten hospital stays.

**Methods:**

We extracted the data of 1488 patients who underwent hip replacement from the hospital case database from November 2017 to May 2021 and used the case–control test to divide the patients into the case group (scheduled ICU admission, 134 cases) and the control group (Not scheduled ICU admission, 213 cases). The general conditions of the patients before surgery, including sex, age, Charlson comorbidity index, laboratory test results, and anaesthesia methods, were collected and used as independent variables. The t test, rank sum test, and X^2^ test were used to analyse and identify significant factors with a *P* < 0.05. Then, these factors were entered into binary logistic regression analysis, and a ROC curve was used to test the efficacy of the regression model.

**Results:**

In the data we collected, 134 patients were planned to be transferred to the ICU, and 213 patients were not transferred to the ICU. The two groups of data were analyzed by logistic regression. We defined the risk factors for preoperative ICU appointment in patients with primary hip arthroplasty, including age. (odds ratio (OR) 1.066, 95% (confidence interval) CI (1.039, 1.093), *P* < 0.001), general anesthesia ( (OR) 1.821, 95%CI (1.165, 2.845), *P* = 0.008), preoperative C-reactive protein ((OR) 1.016, 95%CI (1.010, 1.022), *P* < 0.05), preoperative alanine aminotransferase ((OR) 1.042, 95%CI ((1.016, 1.070)), *P* = 0.002). These were promoting factors for preoperative ICU appointment,and preoperative albumin ((OR) 0.0839, 95%CI (0.792, 0.889)), *P* < 0.05) was a protective factor for ICU appointment.

**Conclusion:**

For patients requiring primary hip replacement. Age, general anesthesia, preoperative C-reactive protein, preoperative alanine aminotransferase and preoperative albumin are the key points of our preoperative assessment. Paying attention to the changes of these indicators will help surgeons assess the patient's condition and contact the ICU in advance.These data can be fully understood by the patients' families, reduce the unnecessary use of medical resources, and optimize perioperative management.

## Introduction

Although hip replacement technology and rapid rehabilitation theories are constantly improving and the number of patients undergoing hip replacement surgery is also significantly increasing each year, there are still minor and major complications that occur after hip replacement [1–3]. After hip replacement, some patients will need an increased level of postoperative monitoring and medical management, including access to intensive care units (ICUs) [4]. Among surgical patients, it is estimated that one in every 30 patients will need intensive care services after total joint replacement [5]. Methods to reduce postoperative complications, the average length of stay and overall cost as well as achieve satisfactory results for the patients and their families are still the focus of many doctors' attention. Abdel-Salam and Sukhonthamarn et al. have analysed the factors influencing a transfer to the ICU after total joint replacement, andAtul F. Kamath studies have reduced the number of ICU transfers and postoperative complications and repeated hospitalization rates through modelling. There are few studies on the risk factors and necessity of preoperative ICU reservation.The purpose of this study was to evaluate the risk factors of analysis of related factors of scheduled ICU before primary hip arthroplasty.

## Material and methods

**Fig. 1:**
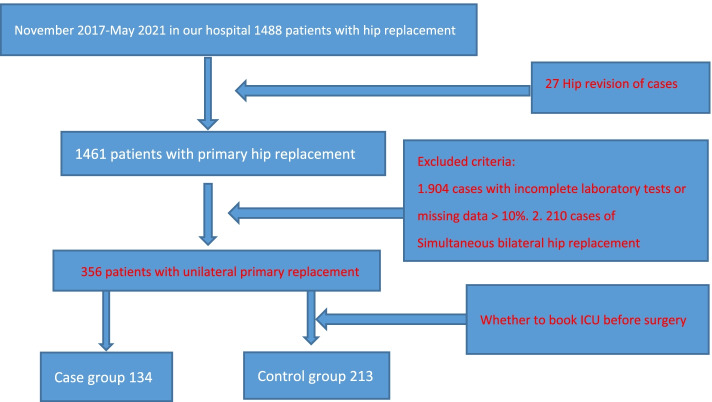
Flow chart of patient screening

After gaining approval from the institutional review committee, we extracted the data of 1488 patients who underwent hip replacement from the hospital case database from November 2017 to May 2021. The medical records were reviewed, and the inclusion criteria were as follows: (1) primary hip replacement; (2) complete and available preoperative general information, preoperative examination results and anaesthesia methods; (3) unilateral surgery; (4) Patients agreed to use their clinical data for research. The methods for handling missing data were as follows: (1) patients with > 10% data missing were excluded; (2) The method of filling in the mean value for some missing important data.According to the methods of a case–control study, there were 134 cases in the case group (scheduled ICU admission) and 213 cases in the control group (unscheduled) admission; see Fig. 1.

Abdel Salam et al. believed that higher C-reactive protein, lower hemoglobin level, higher body mass index, and older age were risk factors for unplanned transfer to ICU after surgery. Sukhonthamarn et al. considered that Charlson comorbidity index, general anesthesia, increased estimated blood loss and decreased preoperative hemoglobin, and increased preoperative glucose level were independently associated factors for increased risk of ICU admission.Sarah E. Rudasill believes that Hypoalbuminemia is associated with increased total direct costs, LOS, and readmissions following primary and revision THA and TKA.There were few laboratory results included in previous studies. Some studies had unilateral observations, such as the effect of preoperative albumin on postoperative transfer to ICU. We hope to include more routine laboratory results to further determine which are the main influencing factors. Therefore, the factors included in the analysis are mainly preoperative blood routine, biochemical examination, coagulation function, C-reactive protein, the general data of patients such as age, CCI, gender and other information.A total of 25 variables comprising preoperative demographic characteristics and laboratory test results were included as independent variables,included age (AGE), Charlson comorbidity index (CCI), preoperative pulse (beat/minute), preoperative anaesthesia (general anaesthesia or spinal epidural anaesthesia), and the following preoperative laboratory parameters: red blood cell (RED) (10^^9^/L), haemoglobin (HP) (g/L), haematocrit (HCT) (%), platelet (PLT) (10^^9^/L), C-reactive protein (CRP) (mg/L), prothrombin time (PT) (seconds), international normalized ratio (INR), thrombin time (TT) (seconds), activated partial thromboplastin time (APTT) (seconds), fibrinogen (FIB) (g/L), K^+^ (mmol/L), total protein (TP) (g/L), albumin (ALB) (g/L), glutamic-oxalacetic transaminase (AST)(U/L), glutamic-pyruvic transaminase (ALT) (U/L), total bilirubin (TBIL) (umol/l), direct bilirubin (DBIL), urea nitrogen (BUN) (mmol/L), serum creatinine (SCR) (umol/L), blood glucose (GLU) (mmol/L).whether the patient was transferred to the ICU was considered the dependent variable.

### Statistical analysis

The measurement data that followed a normal distribution are described as the mean ± standard deviation,measurement data that did not follow a normal distribution are described as the interquartile ranges or as the number of cases (percentage). Independent sample T tests or one-way ANOVA were used for comparisons of measurement data conforming to a normal distribution between groups, and nonparametric tests were used for comparisons of measurement data that did not conform to a normal distribution between groups. In terms of multifactor analysis, a binary logistic regression model was adopted, and a stepwise regression method was used to avoid collinearity problems. In all tests, the difference was statistically significant when *p* < 0.05. ROC curves were used to test the effectiveness of the logistic regression model, and the AUC was calculated. In the regression analysis, *P* < 0.05 was considered statistically significant, and the OR value was analysed to identify the risk factors for preoperative ICU reservation. Statistical data were analysed with SPSS software, version 24.0 (SPSS Inc., Chicago, IL).

## Results

Anaesthesia type, C-reactive protein, haemoglobin, red blood cells, haematocrit, fibrinogen, total protein, albumin, AST, ALT, total bilirubin, direct bilirubin, and blood glucose levels, and age were related with preoperative ICU reservation. Comparison of general data between two groups of patients See Table 1.

**Table 1:** Comparison of general data between two groups of patients

Preoperative reservation of ICU	Case group(134)	Control gourp(213)	P
CCI	1.037 ± 1.334	0.779 ± 1.56	.113
Pulse	85.23 ± 12.55	83.559 ± 12.046	.215
Anesthesia(%)			
General anesthesia	73 (54.5%)	146 (68.5%)	.008
Spinal epidural anesthesia	61 (45.5%)	67 (31.5%)	
CRP	73.12 ± 53.35	29.18 ± 41.59	.000
HP	111.1 ± 21.84	124.03 ± 21.43	.000
RED	3.75 ± 0.83	4.29 ± 0.77	.000
HCT	33.78 ± 6.44	37.66 ± 5.96	.000
PLT	226.93 ± 97.19	244.93 ± 97.45	.094
PT	13.29 ± 3.05	12.91 ± 7.60	.587
INR	1.13 ± 0.28	1.50 ± 6.51	.517
TT	15.60 ± 2.53	15.35 ± 2.05	.316
APTT	34.18 ± 6.18	34.02 ± 5.81	.814
FIB	4.88 ± 1.47	4.38 ± 1.32	.001
K^+^	3.93 ± 0.55	5.46 ± 21.87	.416
TP	62.53 ± 7.60	66.91 ± 6.61	.775
ALB	33.76 ± 5.62	38.80 ± 5.12	.000
AST	28.31 ± 21.31	21.73 ± 8.81	.000
ALT	22.28 ± 15.5	18.83 ± 9.63	.011
TBIL	15.48 ± 8.11	12.91 ± 7.01	.002
DBIL	5.50 ± 3.84	3.85 ± 2.26	.000
BUN	8.35 ± 5.51	7.40 ± 8.24	.238
SCR	118.00 ± 153.41	102.99 ± 118.40	.309
GLU	6.88 ± 2.79	6.01 ± 3.12	.009
Gender (%)			
Female	73 (54.5%)	111 (52.1%)	.668
Man	61(45.5%)	102 (47.9%)	
Age	79.13 ± 11.45	67.90 ± 15.02	.000

*ICU* Intensive care unit, *CCI* Charlson comorbidity index, *CRP* C-reactive protein, *HP* Hemoglobin, *RED* Red blood cell, *HCT* Hematocrit, *PLT* Platelet, *PT* Prothrombin time, *INR* International normalized ratio, *TT* Thrombin time, *APTT* Activated partial thromboplastin timepartial thromboplastin time, *FIB* Fibrinogen, *TP* Total protein, *ALB* Albumin, *AST* Glutamic-oxalacetic transaminase, *ALT* Glutamic-pyruvic transaminase, *TBIL* Total bilirubin, *DBIL* Direct bilirubin, *BUN* Usea nitrogen, *SCR* Serum creatinine, *GLU* Blood glucose

### Binary logistic regression analysis

The demographic data of the patients and the results of the preoperative laboratory tests were statistically analysed. The anaesthesia method, preoperative C-reactive protein, haemoglobin, red blood cells, haematocrit, fibrinogen, total protein, albumin, AST, ALT, total bilirubin, direct bilirubin, blood glucose and age were statistically significant independent variables. Then, binary logistic regression analysis was used to incorporate these meaningful independent variables into the logistic regression analysis with the stepwise regression method. Among them, anaesthesia (general anaesthesia), C-reactive protein, alanine aminotransferase and age were risk factors for preoperative ICU reservation. The use of general anaesthesia and increased preoperative C-reactive protein, alanine aminotransferase and age may increase the probability of preoperative ICU reservation, revealing that these are influencing factors. Albumin is a protective factor against preoperative ICU reservation, and an increase in preoperative albumin did not increase the risk for preoperative ICU reservation.The results are shown in Table 2.

**Table 2:** Binary logic regression analysis of related factors

	Estimate	Std..Error	OR	CI(95%)	P
Anesthesia^a^	0.599	0.228	1.821	(1.165,2.845)	0.008
CRP^a^	0.016	0.003	1.016	(1.010, 1.022)	< 0.05
HP	-0.01	0.034	0.989	(0.925,1.058)	0.752
RED	-0.227	0.192	0.797	(0.547,1.160)	0.236
HCT	0.013	0.42	1.013	(0.934,1.099)	0.752
FIB	-0.062	0.109	0.940	(0.759,1.164)	0.568
TP	-0.025	0.026	0.978	(0.927,1.027)	0.344
ALB^a^	-0.175	0.029	0.0839	(0.792,0.889)	< 0.05
AST	0.001	0.016	1.001	(0.971,1.032)	0.962
ALT^a^	0.040	0.013	1.042	(1.016,1.070)	0.002
TBIL	-0.018	0.031	0.983	(0.924,1.045)	0.573
DBIL	0.053	0.053	1.055	(0.950,1.171)	0.315
GLU	0.033	0.045	1.034	(0.947,1.129)	0.460
AGE^a^	0.064	0.013	1.066	(1.039,1.093)	< 0.001

*CRP* C-reactiveprotein, *HP* Hemoglobin, *RED* Red blood cell, *HCT* Hematocrit, *FIB* Fibrinogen, *TP* Total protein, *ALB* Albumin, *AST* Glutamic-oxalacetic transaminase, *ALT* Glutamic-pyruvic transaminase, *TBIL* Total bilirubin, *DBIL* Direct bilirubin, *GLU* Blood glucose

^a^ Independent prediction of risk factors for preoperative ICU reservation

**Fig. 2:**
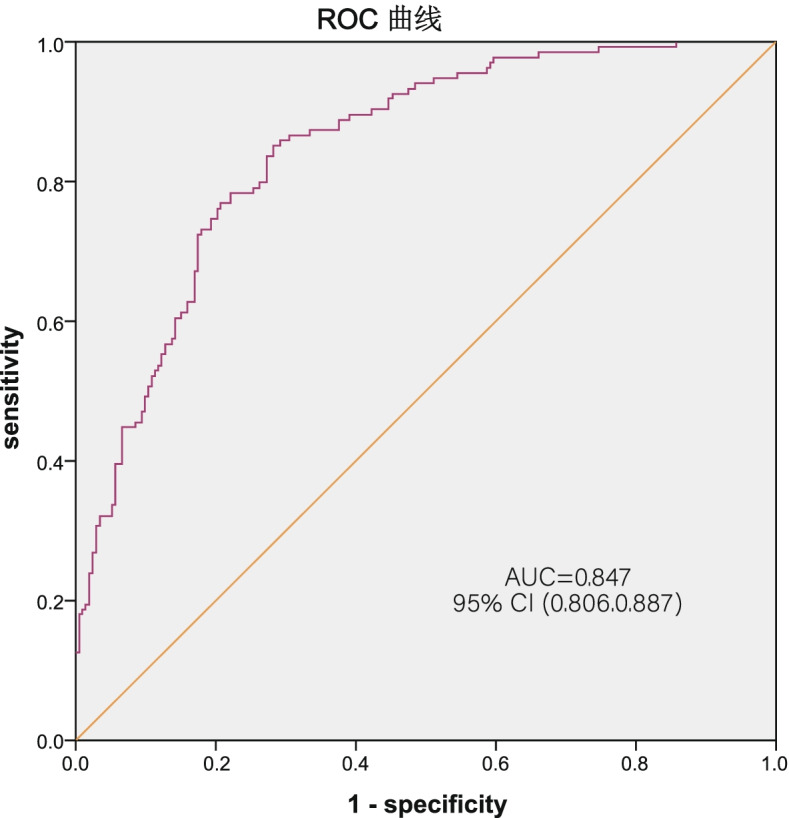
The ROC curve: The area under the ROC curve in the training set was 0.847, and the 95% CI was 0.806–0.887

The performance of the binary logistic regression model was verified by the ROC curve shown in Fig. 2. The area under the ROC curve in the training set was 0.849, and the 95% CI was 0.806–0.8887, indicating that the regression model has good diagnostic value. The closer the area is to 1, the better the performance is.

## Discussion

In clinical practice, our team found that there might be insufficient ICU beds after unplanned transfers to the ICU and poor communication between the operating room and the ICU ward, resulting in patients not being transferred in time. Unplanned transfers to the ICU after surgery increase the psychological burden to the patients and their families as well as the workload of nurses and anaesthesiologists in the operating room due to the lack of preoperative information. In addition, if patients with sudden-onset diseases in a postoperative general ward need to be transferred to the ICU immediately, there may be insufficient human resources in the general ward, leading to untimely treatment and a delay in diagnosing the disease. Moreover, our team believes that unplanned transfers to the ICU after surgery also reflect an incomplete preoperative evaluation by the treatment team. The purpose of this study was to analyse the risk factors for preoperative ICU reservation and explore the necessity of preoperative ICU reservation in primary total hip arthroplasty.

The incidence of severe complications in patients undergoing elective total hip arthroplasty and knee arthroplasty is reported to be 2% -5% [6]. Although surgical techniques and preoperative evaluation techniques are constantly improving, some patients undergoing joint replacement still need to be transferred to the ICU [4]. Through single-factor and logistic regression analysis, we concluded that anaesthesia method, preoperative albumin, C-reactive protein, alanine aminotransferase and age were influencing factors for preoperative ICU reservation.

General anaesthesia was a risk factor for ICU reservation before the initial total hip arthroplasty. In total, 54.5% of the patients in the case group received general anaesthesia. In the logistic regression analysis, the OR of general anaesthesia was 1.821 (95% CI, 1.165, 2.845, *P* = 0.008). Hossam AbdelSalam [7] et al. analysed the factors for an unplanned to transfer to the ICU after joint replacement, and the OR of general anaesthesia was 45.21955 (*P* = 0.044). The OR values in the two studies were > 1. The OR value in this study was lower than that in the study by Hossam AbdelSalam et al., which may be due to the insufficient sample size or different statistical methods. However, the conclusion that general anaesthesia is a risk factor for preoperative ICU reservation was consistent. General anaesthesia has been proven to be more likely to be related to a reduction in oxygen saturation, and the postoperative complication rate may also increase [8]. Compared with associated with general anaesthesia, the incidence and mortality associated with regional anaesthesia may be lower [9]. Patients undergoing primary hip replacement are mostly elderly who have basic diseases such as hypertension, diabetes, coronary heart disease, COPD, and pneumonia. General anaesthesia greatly increases the risk of preoperative ICU reservation, indicating that surgeons and anaesthesiologists should focus on the selection of anaesthesia methods.

Another influencing factor was preoperative albumin. Patients with hypoalbuminemia treated with Total hip arthroplasty (THA)and Total knee arthroplsty(TKA) may have more postoperative complications, including surgical site infection, sepsis, pneumonia, no ambulation for > 48 h and unplanned intubation [10–13]. The benefits of preoperatively identifying serum albumin include its cost and a potential opportunity to preoperative correct malnutrition, which has been proven to improve the postoperative results [6, 14]. In this study, the OR value of preoperative albumin was 0.839 (95% CI, 0.792, 0.889), and preoperative albumin was a protective factor, that is, as the haemoglobin increases, the risk for a scheduled ICU admission will decrease, which is consistent with the observed clinical assessment results.

Another risk factor may affect transfer to the ICU is alanine aminotransferase (ALT), which has not been mentioned in previous literature. This may be related to the fact that ALT is not included as an independent variable in the literature. There were many preoperative examination items included in the analysis in this study, including almost all haematological examination variables. An increased preoperative ALT indicates abnormal liver function preoperatively. It is necessary to further analyse hepatitis B surface antibodies or antigens in combination with other risk indicators to comprehensively consider whether patients need an ICU reservation and whether clinical interventions or a temporary surgical intervention is necessary. In this study, the OR of ALT as a risk factor was 1.042 (*P* = 0.01, 95% CI, 1.016, 1.070), indicating that ALT is a risk factor for preoperative ICU reservation. As the ALT increases, the probability of preoperative ICU reservation increases, indicating that clinicians should consider ALT in the analysis of whether a preoperative ICU reservation is needed.

Age is usually included in the patient assessment as a risk factor in clinical work, and elderly patients are more likely to undergo hip replacement surgery [15], and these patients have an increased risk for postoperative complications [16]. In the study by Mantilla et al., old age was found to be the strongest predictor of major postoperative complications [16]. In this study, age was a risk factor for preoperative ICU reservation (OR = 1.064, 95% CI, 1.038, 1.091), as in the literature. With improvements in quality of life, the number of elderly patients undergoing hip replacement is gradually increasing. Elderly patients often have basic diseases, poor nutritional status, and weak immune responses. This study suggests that elderly patients should be fully evaluated before surgery, and basic diseases such as malnutrition and anaemia should be corrected early. Preoperative ICU reservation has certain advantages in reducing postoperative complications and delirium after anaesthesia.

C-reactive protein is a nonspecific marker of inflammation and is often used as a marker of periprosthetic infection [7]. Increases in preoperative C-reactive protein will lead to increases in the probability of postoperative infection, and infections after joint replacement can be catastrophic. In our study, a traumatic stress response led to a high preoperative C response protein in patients with femoral neck and femoral shaft fractures. In the study by Sukhonthamarn et al., C-reactive protein was not an independent influencing factor for postoperative ICU transfer in the multivariate logistic regression analysis, while in the study by AbdelSalam et al., C-reactive protein was an independent influencing factor; this may be related to the differences in the statistical methods used. In our study, the OR value of C-reactive protein was 1.016 (95% CI, 1.010, 1.022), which was basically consistent with that in the study by AbdelSalam et al. This shows that an increase in C-reactive protein before surgery is an important consideration for surgeons. It is necessary to pay attention to whether patients need ICU reservations to reduce the occurrence of postoperative complications. The increase in C-reactive protein is due to a post-traumatic stress response or recessive infection, such as hypostatic pneumonia or urinary tract infection.

There are also many limitations in this study. This study is based on an analysis of medical records and lacks forward-looking analysis and prediction model construction. We will continue to improve upon these points in subsequent studies. Patients underling hip replacement are often elderly, have underlying diseases and have unstable internal environments. Patients at high risk before surgery have previously been transferred to the ICU or to general wards after surgery. Patients with sudden-onset diseases need to be transferred to the ICU. However, there may be insufficient ICU beds and medical personnel in both schemes. The patients’ and their families may find it difficult to accept when they are suddenly informed that they need to be transferred to the ICU after the operation. After a period of exploration, we implemented a scheme of fully evaluating the patient’s condition before the operation and making an ICU reservation in advance to help most patients understand the decision and reduced the psychological pressure to the patients and their families. However, most of the patients scheduled for ICU admission in this study were only in the ICU for one day, and most did not need intubation or ventilation. After the first postoperative day, the patients returned to the general ward after their condition was stable, which had a positive effect on hospitalization time, cost, and surgical safety, reducing postoperative complications and optimizing the medical resource scheme. This study suggests that this program can be adapted based on each hospital and each ICU configuration, combined with the preoperative patient assessment and enable individualized implementation. This approach can also be used for preoperative evaluations before outpatient hip surgery. In summary, this study suggests that preoperative ICU reservation is necessary and clinically significant in primary total hip arthroplasty.

## Data Availability

The datasets used and analysed during the current study available from the corresponding author on reasonable request.

## Abbreviations

ICU: Intensive care unit
ROC: Receiver operating characteristic curve
CCI: Charlson comorbidity index
RED: Red blood cell
HP: Hemoglobin
HCT: Hematocrit
PLT: Platelet
CRP: C-reactive protein
PT: Prothrombin time
INR: International normalized ratio
TT: Thrombin time
APTT: Activated partial thromboplastin timepartial thromboplastin time
FIB: Fibrinogen
TP: Total protein
ALB: Albumin
AST: Glutamic-oxalacetic transaminase
ALT: Glutamic-pyruvic transaminase
TBIL: Total bilirubin
DBIL: Direct bilirubin
BUN: Usea nitrogen
SCR: Serum creatinine
GLU: Blood glucose
